# Book Review: Self and Social Identity in Educational Contexts

**DOI:** 10.3389/fpsyg.2017.01775

**Published:** 2017-10-10

**Authors:** Dean P. McDonnell, Laura M. Griffin

**Affiliations:** ^1^School of Education, Trinity College Dublin, Dublin, Ireland; ^2^Department of Technology and Psychology, Dún Laoghaire Institute of Art, Design, and Technology, Dublin, Ireland

**Keywords:** social identity, educational practice, psychology of education, practice based research, educational context

## A 30 second review

Books that are insightful, thoughtful provoking, and written in a way that is accessible to multiple reader types are hard to come by; this, we believe, is one of them. Mavor, Platow, and Bizumic (2017) have provided us with a book that is equally as important in education as it is in psychology. We would strongly encourage academics, educators, and students, who are interested in extending their knowledge of educational practice and research, to keep a copy of this book on their desk for easy access!

## Full review

The “self” and “social identity” are two core principles that have long fascinated psychologists and sociologists. This broad list of interested academics, researchers, and practitioners ranges from Jung, who aimed to understand the “self” through the interpretation of the unconscious and symbolism (Jung and Shamdasani, 2010), to Tajfel and Turner (1979), who proposed that an individual's perception of who they are is heavily influenced by their membership(s) of a group (or groups). Tajfel and Turner (1986) suggest that, because there are key differences between an individual's personal and social identity, the differences are determined by perceived experiences within interpersonal and group situations. While some theorists have recognized that social identity theory can be limited in its differentiation of groups (Turner et al., 1987; Brown, 2000), other academics across the social sciences have attested to the use of social identity theory in generating hypotheses for the study of group behavior (Huddy, 2001; Bradford, 2014; Jenkins, 2014). While the above is, undoubtedly, a non-exhaustive list of those currently working within the area of “self” and “social identity,” Mavor, Platow, and Bizumic's book “*Self and Social Identity in Educational Contexts*” (2017) utilizes social identity theory (Tajfel and Turner, 1979) combined with self-categorization (Turner et al., 1987), referred throughout as the “social identity approach,” to highlight the importance of developing “*new and creative forms of practice in educational settings*” (p. i). In addition to the wealth of knowledge and experience of 44 academics, researchers, and practitioners, this book is viewed as an invitation for others to explore the “*social and psychological processes by which learners' personal and social self-concepts shape and enhance learning and teaching*” (p. i).

While this book is targeting *advanced* students and educational researchers across the social sciences, the introductory chapters are written in a way that, one would argue, could certainly appeal to a much wider audience. The first Chapters (Ch1 and Ch2) are given the monumental task of introducing what the contextualizing of the self and social identity within education looks like to paint a picture of how the book will progress. The challenge of this chapter, as is identified, is the diversity in the later chapters; how does one clearly articulate so many specialized areas of social identity and self-categorization? Taking a broadened operational approach, the authors note structuring the chapters by context, stages, and mechanisms associated with the identity and aid the reader by offering themes in which readers should view each chapter: (i) Promoting Change, (ii) Managing Conflict, and (iii) Facilitating Change. The way in which the various sections of this book are placed guides the reader along a linear pathway of information that concludes with the applied chapters (Ch16, Ch17, and Ch18) and concluding Chapter (Ch19). Among the central Chapters “Between the Classroom and Beyond” (Ch5, Ch6, Ch7, and Ch8) and “Student Life” (Ch9, Ch10, and Ch11) for example, the authors provide insight into how life transitions can involve moments of change regarding social identity and that the personal self can be affected in this process. There is evidence and arguments that call for a type of reformation of educational practice to account student engagement extending past quality teaching and adopting methods that enhance group and classroom cohesiveness and dynamics.

It is evident, in the way this book is written, that most authors are both academics and practitioners. This interplay of two worlds provides readers with a unique insight into research and theory that is practice-orientated and succeeds in “*opening and explaining novel integrations between psychology and education for the mutual benefit of both*” (p. 16). Although the theoretical aspects are explained in intricate detail, they are done so in a way that is comprehensive and understandable. Initially, there was some concern regarding the specificity of certain chapters, such as Chapter 6 which focuses on “Insights and Applications from Medical Education” (as this is not necessarily a personal research interest), but found that, after this review, these chapters had a higher number of post-it notes ranging from “*interesting*” and “*never thought of that*” to “*that's incredible*” and “*oooh, look this up*.”

As mentioned above, this book appeals on multiple levels. One of the resounding statements comes from Chapter 12 to 15, “Approaches to Learning and Academic Performance,” where the authors highlight that focusing on the ***way*** students learn is no longer as important as ***how*** students learn; that there is need to account for the holistic learner in terms of ontology and epistemology. The book is addressing and highlighting critical research areas within the combined fields of education and psychology. While some of the chapter foci can often be considered niche, such as “bullying,” “group identity,” or “socio-economic status,” the applied nature of the writing, combined with the wealth of experience from the authors, packages the content into a digestible one-inch thick book.

## Author contributions

This review was completed in a number of steps. First, both authors read and independently reviewed this book. Second, using an interview style process, the lead author (DM) asked the second author (LG) a series of questions related to the content, quality, and style, of each Chapter within the book. Finally, the lead author (DM) compiled both reviews, compared these reviews against the interview data, and wrote the final document.

### Conflict of interest statement

The authors declare that the research was conducted in the absence of any commercial or financial relationships that could be construed as a potential conflict of interest.
